# Depressive symptoms in response to COVID-19 and lockdown: a cross-sectional study on the Italian population

**DOI:** 10.1038/s41598-020-79850-6

**Published:** 2020-12-31

**Authors:** Marco Delmastro, Giorgia Zamariola

**Affiliations:** 1 Autorità per le Garanzie nelle Comunicazioni (AGCOM), Via Isonzo 21/b, Roma - Centro Direzionale Isola B5, Napoli, Italy; 2 Alma Mater Studiorum, Università di Bologna, Via Zamboni, 33, Bologna, Italy

**Keywords:** Diseases, Health care, Risk factors

## Abstract

The COVID-19 pandemic and the lockdown orders adopted to prevent the spread of the disease had a huge impact on a personal, social, and economic level for the world population. In Europe, Italy was one of the frontrunner countries dealing with an emergency that significantly affected people’s lives. Previous research on the psychological impact of the pandemic revealed an increase in anxiety, depression, and feelings of distress; however, these studies were conducted on non-representative samples of the population reached through social media channels, a method that is likely to lead to many forms of statistical and methodological bias. For the first time to our knowledge, we assessed the psychological impact of COVID-19 on 6700 Italian individuals, representative of the Italian population in terms of age, gender, and geographical areas revealing higher scores of depressive symptoms in females, younger adults, people reporting professional uncertainty and lower socio-economic status. A positive correlation was also found for individuals living alone, those who could not leave home for going to work, and people with a case of COVID-19 in the family, whereas the region of residence was not a significant predictor of depressive symptoms. These findings underline the importance of considering the psychological effects of COVID-19 and providing support to individuals seeking mental health care.

## Introduction

On 31 December 2019, the World Health Organization (WHO) received information of cases of pneumonia of unknown cause in Wuhan City, China. The cause was identified by Chinese authorities as being a novel coronavirus afterwards named the “COVID-19 virus”. Due to the rapid increase in the number of cases outside China, on 11 March 2020, WHO Director-General announced that the outbreak could be characterized as a pandemic.

After China, Italy was the first European country to report a case of death due to COVID-19 dated on 21 February 2020 in Lombardy, followed by a series of outbreaks in the Northern regions and a rapid increase of deaths and cases of infection. In response to this emergency, Italy was the first country in Europe to adopt restrictive physical and social distancing measures to limit the spread of the disease which led to a full lockdown of the entire country from 9 March 2020 until 3 May 2020 (so-called “phase 1”), and other measures, such as the prohibition of individual movements outside people's region of domicile, until 2 June (“phase 2”; for more information see COVID-19 Health System Response Monitor^1^). Since the beginning of the emergency there have been 233,515 confirmed cases of COVID-19 with 33,530 deaths reported (as of 2 June 2020, data retrieved from the Italian Ministry of Health).

The period of lockdown has been characterised by travel restrictions and the mandatory closure of schools, nonessential commercial activities and industries. People were requested to stay at home and socially isolate themselves to prevent the spread. Such an experience has the potential to influence people’s mental state evoking fear of contagion, worry about disease and death, in addition to anxiety due to health and economic uncertainties^2^. Severe pandemic lockdowns and quarantine are a further source of distress since individuals are confined, socially isolated, might lose their income, and their activities are restricted^3^. This situation is worsened by an “infodemic”, i.e., an overabundance of information, some of which can be misleading or even harmful^4^.

In the next sections, we will provide an overview of previous international and national (Italian) studies on the psychological impact of COVID-19 on the population. We will then present our study which was conducted on a sample of the Italian population exploring the psychological effects of COVID-19 pandemic and lockdown. While previous research adopted a convenient sampling strategy administering the questionnaire through social media channels and snowballing technique, our study relied on a random and representative sample which avoids recruitment bias (i.e., sample selection, hence biases in statistical results).

### Previous international studies

Recent studies have shown that the COVID-19 pandemic affected the mental health. One recent narrative review^5^ comprises 28 articles addressing the issue of mental health revealed the presence of symptoms of anxiety, depression, and self-reported stress associated with disturbed sleep in response to the pandemic. These articles include samples from China, Iran, Canada, Brazil, Singapore, India, and Japan. The results showed that variables such as female gender, being a student, having symptoms suggestive of COVID-19, and poor perceived health were associated with higher rates of anxiety and depression. Other characteristics that contributed to stress and mental morbidity were unpredictability, uncertainty, seriousness of the disease, misinformation, and social isolation.

Another review^6^ pointed out that maladaptive behaviours, emotional distress, and defensive responses are psychological reactions that can be experienced during a pandemic. More specifically, data reported the prevalence of significant post-traumatic stress symptoms and anxiety.

Two studies compared mental health pre- and post-confinement. The first one^7^ revealed a change in mood and feelings as measured by the Short Mood and Feelings Questionnaire (SMFQ). As reported by the authors, the total score increased significantly by 44.9% “during” compared to “before” home confinement (*p* < 0.001, *d* = 0.44). The second study^8^ compared measures pre- and post-lockdown showing an increase in psychological distress after the lockdown. However, higher levels of sense of community and resilience were also found, providing support to the presence of some positive outcomes related to the challenges faced during the pandemic.

### Previous studies conducted in Italy

A study on a sample of 18,147 individuals from the Italian population^9^ showed high rates of post-traumatic stress symptoms (37.14% of the sample), depression (17.3% of the sample), anxiety (20.8% of the sample), sleep disorders (7.3% of the sample), perceived stress (21.9% respondents scoring above the threshold) reported during the lockdown, with females expressing higher impact on their mental health. Other risk factors identified were being under quarantine, having a significant one deceased by COVID-19, working discontinuity, or experiencing other stressful events (i.e. working, financial, relationship or housing problems) linked to the pandemic or lockdown measures. The data were collected between March 27th and April 6th 2020 using an on-line questionnaire spread throughout the internet, using sponsored social network advertisement together with a snowball recruiting technique.

Another web-based cross-sectional survey on 2291 Italian individuals was conducted through different platforms and mainstream social-media and enabled from March 18th to April 2nd^10^. The results showed a relationship between COVID-19 spread and feelings of anxiety (32.1% of the respondents), psychological distress (41.8% of the respondents), sleep disorders (57.1% of the respondents) and symptoms of post-traumatic stress disorders (7.6% of the respondents). A similar research^11^ using the same sample as the study previously mentioned^10^ revealed that being of younger age, student, female, and having had a direct contact with COVID-19 infection were associated with a greater psychological impact on psychopathological dimensions such as anxiety, distress, and sleep disturbance.

As highlighted in another recent research on 2766 Italian participants^12^, female gender, negative affect, and detachment were related to higher levels of psychological distress. Depression levels varied, with 67.3% of respondents reporting an average level, 17% belonging to the high range, and 15.4% in the extremely high range. Higher depression and stress levels were found in people with an acquaintance infected with COVID-19 and in individuals with a history of stressful situations and medical problems. Other factors affecting anxiety and stress levels were having a family member infected with COVID-19, being young in age and needing to leave one’s domicile to go to work. In this case, data were collected over 5 days (18–22 March 2020). The questionnaire was administered cross-sectionally using an online survey disseminated through the main means of communication and social networks.

Additional research on a sample of 1035 Italian participants identified moderating variables that have an impact on reported mental health during the lockdown^13^. A total of 27.0% of participants reported mild to moderate COVID-19-related peritraumatic distress and 1.6% reached severe symptomatology. Regarding anxiety symptoms, 12.3% and 3.0% of the sample reported moderate and severe levels of anxiety, respectively. For what concern depressive symptomatology, 15.5% showed moderate levels and 6.2% severe depressive symptomatology. This study included a measure of psychological flexibility described as the ability to adapt to changing situation with a resilient attitude. The results of the survey revealed that psychological flexibility is a protective factor which helps to mitigate the negative effects of the pandemic and lockdown, such as depression, anxiety, and distress. As well as previous studies, this research was conducted through an online survey during the Italian mandatory lockdown. Participants were recruited using social media and a snowballing procedure.

### The present study

The aim of the present research was to explore the effects of COVID-19 on mental health. To this end, the SMFQ^14^ was administered to a random and representative sample of the Italian population. Given the sample age range (16–65+), the SMFQ was chosen for its simplicity and ease of understanding. As previously stated, to our knowledge, this is the first study conducted on a random and representative sample of the Italian population.

The study was conducted right after the lockdown phase, in June 2020 (from the 4th of June to the 19th of June), in order to collect the immediate reactions to the emergency. Indeed, we carried out the survey as soon as the lockdown phase was just over (i.e. “Phase 1”), with the re-opening of manufacturing industries and construction sites and the restart of movements across Italian regions (from 3rd of June). This timeframe appears ideal to conduct a field analysis because the whole lockdown phase had just ended, the citizens' memory of the lockdown period was still intact and the organisation of such a complex survey was feasible.

Socio-demographics were also collected to analyse which kind of moderators might represent risk or protective factors for depressive symptoms. Socio-demographics variables included age, gender, education, and socio-economic status. Information on profession and household (living alone or not) and working (still going out to reach workplace or not) conditions were also gathered. Lastly, questions on the municipality of residence and the presence of a case of COVID-19 in the family were included.

## Method

### Sample characteristics

In this work, we focused on young adults (16–24) and adults (25+) so as to proceed with a self-administered questionnaire that was completed by the sampled respondents (younger individuals would have needed the direct assistance of their parents, making the filling in of the questionnaire very cumbersome). The final sample was composed of 6692 Italian individuals, representative of the Italian population in terms of age, gender, and geographical area. More specifically, the sample design and the stratification were based on the following variables: (1) age (7 age groups: 16–17; 18–24; 25–34; 35–44; 45–54; 55–64: 65 +); (2) gender, (3) geographical breakdown (all Italian regions and size of the residential community; 7 classes), (4) education (2 classes: graduates and non-graduates).

### Measures

To evaluate the effect of the pandemic on mood and feelings of the Italian population a psychometric approach was adopted. Due to the special nature of the period which prevented to conduct experimental studies meeting participants face-to-face, a psychometric self-reporting methodology was chosen. We adopted the SMFQ^14^ which includes 13 items indicating how much individuals have felt mentally distressed during the last few weeks (e.g., “I felt miserable or unhappy”, “I didn’t enjoy anything at all”, I found it hard to think properly or concentrate”). The items include general statements related to mood and feelings and might indicate the presence of clinically relevant disorders such as depression and anxiety. The answers are given on a three-point scale where respondents are asked to decide if the statements are “true”, “sometimes true”, or “not true”. Scoring of the SMFQ is obtained by summing together the point values of responses for each item. The response choices and their designated point values are as follows: “not true” = 0 points, “sometimes true” = 1 point, “true” = 2 points. Higher scores on the SMFQ indicate more severe depressive symptoms. The range of scores on the SMFQ varies from 0 to 26. A score of 12 or higher may indicate the presence of clinically relevant depressive symptoms in the respondent^15^. Suggested cut-offs on the SMFQ self-report have been divergent with cut-offs ranging from 4–5 in studies with just fair AUC and younger subjects to a high of 10–12 in studies with good AUCs and older subjects^16^.

The SMFQ has been validated with children and young people aged between 6 and 19, however it has been shown^17^ that it is a useful and valid diagnostic tool for studying mental health within a community-based sample in late adolescence and that it relates well to an adult measure of depression, namely the Clinical Interview Schedule-Revised form (CIS-R^18^). As highlighted in the Introduction, the questionnaire has been also recently used in a sample of adults to measure the emotional impact of COVID-19^7^. The SMFQ has a number of important (psychometric and implementation) features (i.e. internal consistency, test–retest reliability, validity, sensitivity to change as to the former, and brevity, availability, ease of scoring as to the latter), thus making it a useful tool for analysing mood and feelings attitude, especially during a pandemic when other methodologies such as experimental lab studies present severe and objective constraints.

### Procedure

The field data collection was conducted in June 2020 (from the 4th of June to the 19th of June) with a mixed technique CATI (Computer Assisted Telephone Interviewing) and CAWI, (Computer Assisted Web Interviewing) as to limit any risks in terms of sample’s distortion and self-selection. Both types of questionnaires were headed by a detailed description (either by phone from the interviewer or via the web before the start of the questions) of the research aims and objectives. In this context, the interviewees were informed that all their personal data would be acquired anonymously and in full compliance with privacy laws.

All methods were carried out in accordance with the Declaration of Helsinki. The experiment was approved by the Ethics Committee of Autorità per le Garanzie nelle Comunicazioni. Informed consent was obtained from all participants and from a parent and/or legal guardian for participants under 18.

Data analysis was performed using Stata Statistical Software and R.

Even if the SMFQ has been used in the past on samples of adults^7^, it has been mainly validated for children and young adults^17^, therefore in our analysis we separated young adults (aged between 16 and 24) from adults (25+). Table 1 illustrates the age distribution (weighted and not weighted) of our sample and offers a comparison with the Italian population, showing very similar values.

**Table 1:** Population: sample (weighted and not weighted) vs. Italian population.

	Sample population		Italian population
	N	% (weighted)	%
Total (16 +)	6692	100.0%	100.0%
Young adults (16–24)	666	10.0% (10.3%)	10.2%
Adults (25 +)	6026	90.0% (89.7%)	89.8%

As a preliminary remark regarding data consistency, it should be noted that Cronbach’s alpha—i.e., a measure of the reliability of the questionnaire—is equal to 0.91 for the overall sample, 0.90 for the young adult group and 0.91 for the adult group, where alpha 0.90 or above is considered excellent for internal consistency reliability of the SMFQ^15,16^. We added to these variables official COVID-19 statistics in order to disentangle the role played by the spatial variability of the pandemic diffusion on individual mood. We used the official data released every day at 6 PM (UTC + 1 h) by the “Dipartimento della Protezione Civile” (the National Department of Civil Protection of the Presidency of the Council of Ministers) and archived on GitHub (see https://github.com/pcm-dpc/COVID-19). At times, data may just be a proxy of the actual state variables. In particular, the number of confirmed COVID-19 cases (i.e. new COVID-19 positives and total COVID-19 positives) depends on the effort being devoted to finding new positive cases. In addition, deaths statistics for Italy include coronavirus victims who died either in hospital or outside and were tested before or after dying. Regardless of their goodness and accuracy, these statistics are those that have been most widely disseminated by health institutions and the news outlets, thus potentially influencing the mood and feelings of Italian citizens.

## Results

### Preliminary demographic analyses

**Figure 1:**
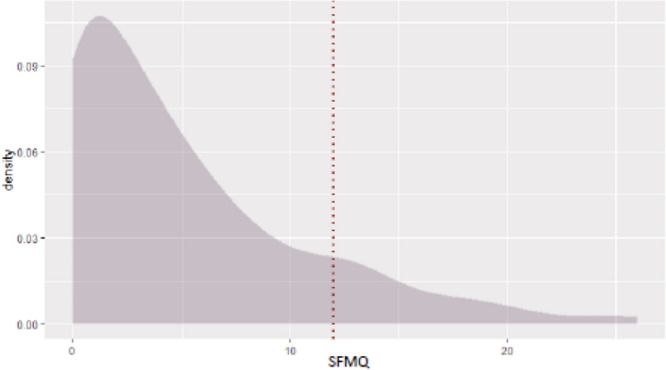
Distribution of SMFQ Scores on total population.

**Figure 2:**
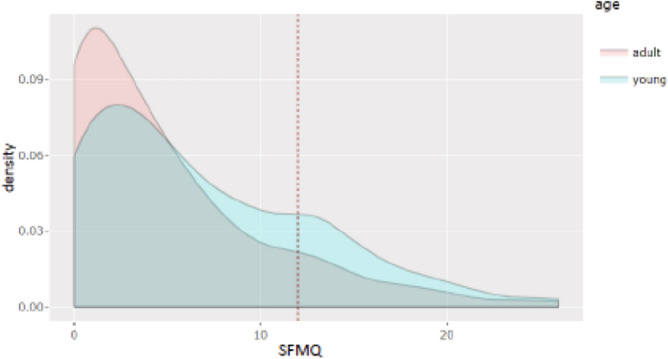
Distribution of SMFQ Scores on young adult (16–24) and adult (25 +) groups.

The first step was to analyse the distribution of the SMFQ score among Italian population, just after the lockdown period, i.e., in June 2020 (see Fig. 1). As we would expect, the total distribution of SMFQ score is asymmetric and skewed to the right (also by a skewness test), with mean at 5.2. It presents a mode near 0, and a slight bump around 12. If we define a cut-off at 12 that may indicate the presence of depressive symptoms in the respondent^15^, then, at June 2020, 14.4% of the Italian population lied above this threshold. In Fig. 2, we compare the score distribution of the two age groups: young adults (16–24) vs. adults (25+). Both distributions follow a similar pattern (skew to the right with a mode near 0 and a long tail thereafter), however the scores across groups age do not have the same distribution function (nonparametric statistical tests (i.e. Kolgomorov-Smirnov test, Wilcoxon rank-sum test and Epps-Singleton test) confirm that the two samples are not drawn from the same distribution function. For this reason, the econometric analysis is also run for the two age groups separately (see Table 6)). In particular, the mean score of the two groups is significantly different (7.04 for young adults and 4.97 for adults), and the probability of scoring a higher value than the cut-off (SMFQ ≥ 12) is significantly higher for the youngest (24.17% vs. 13.33%; Table 2).

**Table 2:** SMFQ score for sample population, and by age groups (weighted and not weighted).

Population	Average (weighted)	% ≥ 12 (cut-off) (weighted)	Minimum	Maximum
Total	5.18 (5.21)	14.41 (14.83)	0	26
Young adults (16–24)	7.04 (7.08)	24.17 (24.91)	0	26
Adults (25+)	4.97 (5.00)	13.33 (13.68)	0	26

### Main analyses to explore predictors of depressive symptoms

The second step was to run estimates of count data models on the overall population, correlating the SMFQ score to some major determinants. Following the existing literature (see above), we included socio-demographic (age and gender), economic (unemployed, lay-off and poor) and context (living alone, size of the municipality of residence) variables (we have also conducted additional analysis including interaction terms into the estimates (e.g. Gender * COVID-19; Gender * No_Lockdown), however they did not display any significant effect, thus confirming the direct effect of explanatory factors). Their operative description and summary statistics are reported in Tables 3 and 4 (see also Table A.1 for descriptive statistics for the two age groups: young adults and adults). Given the characteristic of the dependent variable (i.e. the SMFQ score, which is an integer which lies between 0 and 26) a (Poisson) count model with double censoring (left at 0 and right at 26) has been estimated (In the Appendix Table A.2, we present also results of linear models). Model (1) in Table 5 shows results of the count model with aforementioned “traditional” explanatory variables. Younger individuals are confirmed to be more exposed to negative mood indicative of mental distress (the coefficient of age is negative and significant at 99%), as well as women (coefficient 0.212 and std. dev. 0.0286). This means that younger adults and women are more vulnerable when it comes to the onset of depressive symptoms in response to the pandemic. A negative economic condition significantly affects mental health in terms of future prospect (unemployed: 0.152 and std. dev. 0.0497), absolute value (poor: 0.217 and std. dev. 0.0394) and, above all, professional uncertainty (lay-off: 0.482 and std. dev. 0.0658) (significantly, uncertainty displays (by t-tests) a significantly higher effect than a negative economic status). This result stresses the importance of considering also economic vulnerability which the pandemic and lockdown affected impacting people of lower socio-economic status. Loneliness is also significantly correlated with higher values of SMFQ, both in terms of family nucleus (i.e., living alone: 0.166 and std. dev. 0.0398) and social context (i.e. size of the municipality of residence (note that this variable is (negative and) significant in a logarithmic but not in a linear form, meaning that living in small and very small towns mostly influences (positively) the probability of scoring a high value of SMSQ): − 0.0235 and std. dev. 0.0071). The lockdown aggravated the condition of people living alone removing their opportunities to gather with closed ones and limiting the moments of social exchange and sharing which resulted in developing depressive symptoms.

**Table 3:** Summary statistics of independent variables (weighted and not weighted).

Variable	Description	N	Avg. (weighted)	Std. dv	Minimum	Maximum
Gender (female)	1 if female; 0 otherwise	6692	.524 (.517)	.499	0	1
Age	Age (number of years)	6692	50.489 (50.323)	17.928	16	96
Unemployed	1 if unemployed; 0 oth	6692	.066 (.076)	.249	0	1
Lay-off	1 if a lay-off; 0 otherwise	6692	.025 (.029)	.155	0	1
Poor	1 if poor^(^*^)^; 0 otherwise	6692	.110 (.123)	.313	0	1
Living alone	1 if she/he lives alone; 0 otherwise	6692	.154 (.149)	.360	0	1
COVID-19	1 if a COVID-19 case in her/his family; 0 otherwise	6643	.076 (.076)	.265	0	1
No lockdown	1 if he/she has kept working from workplace (during lockdown); 0 if she/he experienced home lockdown	6643	.102 (.109)	.302	0	1

^(^*^)^“Poor” is defined from a 5-point Likert scale as the worst economic condition, in which the individual experiences severe economic difficulties, lacking sufficient money to live at a normal standard (in 2019, the Italian Statistical Office has estimated that 7.7% of Italian individuals could be regarded as poor; however, this values has sharply increased in 2020 due to the economic crisis related to the COVID-19 pandemic).

**Table 4:** Summary statistics of geographical variables (at the 31st of May 2020).

Variable	N	Average	Minimum	Maximum
Population of the municipality of residence^(^*^)^	2172	19,715	100	2,761,477
Population of the county of residence	110	550,048	56,938	4,300,000
Population of the region of residence	20	3,013,808	125,666	1.00e + 07
Number of COVID-19 cases (county)	110	2088	22	23,076
Number of COVID-19 deaths (region)	20	1671	22	16,112

^(^*^)^ In Italy there are 7,982 municipalities; sample individuals live in 2,172 of them.

*Sources*: Italian Statistical Office (ISTAT) and Italian Ministry of Health.

**Table 5:** Result of econometric models on the SMFQ score.

Variables	(1)	(2)	(3)	(4)	(5)	(6)
	regr_1	regr_2	regr_3	regr_4	regr_5	regr_6
	mfq	mfq	mfq	mfq	mfq	Mfq
Gender (female)	0.212***	0.238***	0.238***	0.240***	0.239***	0.240***
	(0.0286)	(0.0302)	(0.0303)	(0.0297)	(0.0299)	(0.0298)
Age	− 0.0108***	− 0.00985***	− 0.00986***	− 0.00984***	− 0.00985***	− 0.00983***
	(0.000828)	(0.000838)	(0.000839)	(0.000839)	(0.000839)	(0.000839)
Unemployed	0.152***	0.133***	0.133***	0.126**	0.128**	0.127**
	(0.0497)	(0.0503)	(0.0505)	(0.0496)	(0.0500)	(0.0498)
Lay-off	0.481***	0.316***	0.316***	0.321***	0.315***	0.319***
	(0.0658)	(0.0802)	(0.0805)	(0.0845)	(0.0823)	(0.0844)
Poor	0.217***	0.219***	0.218***	0.213***	0.216***	0.215***
	(0.0394)	(0.0425)	(0.0426)	(0.0431)	(0.0427)	(0.0430)
Living alone	0.166***	0.192***	0.192***	0.195***	0.194***	0.195***
	(0.0398)	(0.0409)	(0.0408)	(0.0410)	(0.0409)	(0.0410)
Pop. municipality (log)	− 0.0235***	− 0.0203***	− 0.0200***	− 0.0217***	− 0.0206***	− 0.0209***
	(0.00712)	(0.00718)	(0.00745)	(0.00723)	(0.00720)	(0.00721)
COVID-19		0.733***	0.734***	0.748***	0.741***	0.744***
		(0.0562)	(0.0570)	(0.0531)	(0.0544)	(0.0537)
No lockdown		− 0.123**	− 0.123**	− 0.122**	− 0.123**	− 0.122**
		(0.0551)	(0.0549)	(0.0540)	(0.0542)	(0.0543)
# COVID-19 cases (county)			− 4.18e−07			
			(2.46e−06)			
% COVID-19 cases pop. county				− 7.638		
				(4.297)		
# COVID-19 deaths (region)					− 3.24e−06	
					(2.60e−06)	
% COVID-19 deaths pop region						-40.10
						(26.51)
Constant	2.199***	2.054***	2.052***	2.095***	2.067***	2.080***
	(0.0872)	(0.0904)	(0.0910)	(0.0931)	(0.0909)	(0.0920)
Observations	6692	6643	6643	6643	6643	6643

^(^*^)^ Note: The estimates in the table refer to Poisson count models with double censoring (left at 0, right at 26), sample weights and robust standard errors. The dependent variable is the SMFQ score. For each variable, the coefficient, the standard error (in parentheses), and the level of significance are reported as follows: *** significant at 99%; ** significant at 95%.

**Figure 3:**
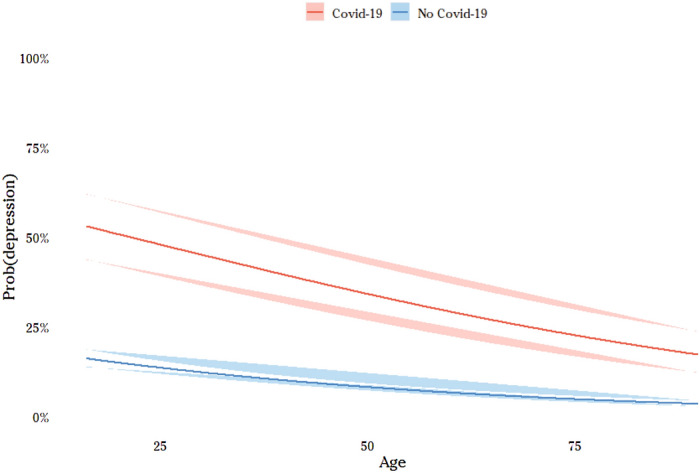
The effect of COVID-19 on the probability of depression (cut-off ≥ 12). ^(*)^ Note: effects calculated on the basis of the probit model of Table A.3 (age interval: 16–95). Confidence interval at 99%.

**Figure 4:**
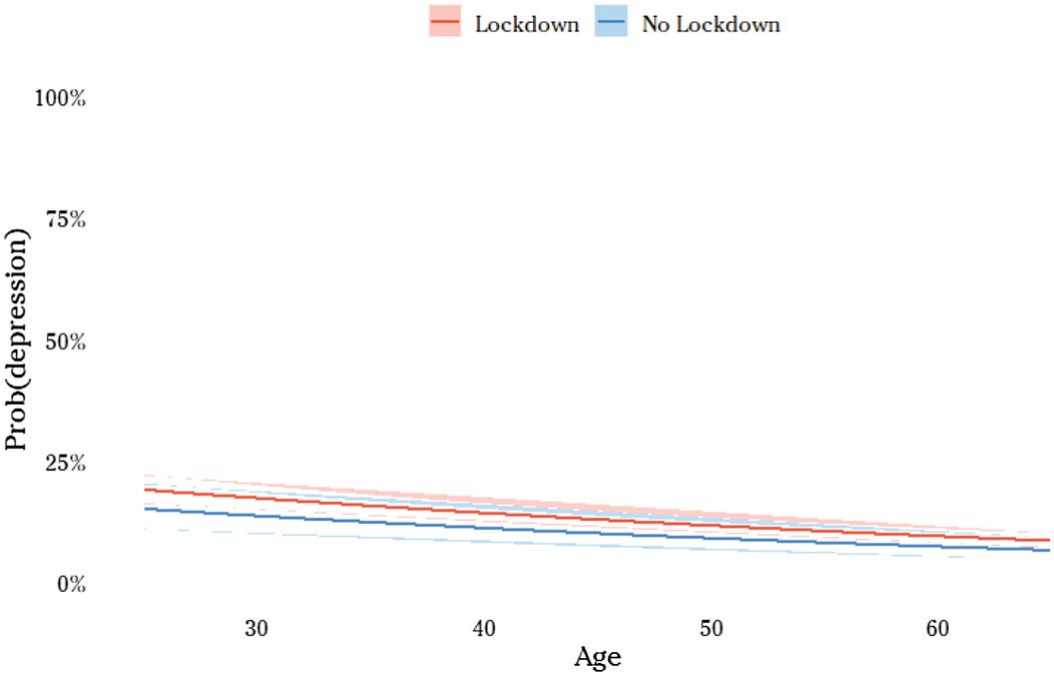
The effect of lockdown on the probability of depression (cut-off ≥ 12). ^(*)^ Note: effects calculated on the basis of the probit model of Table A.3 (age interval: 25–65). Confidence interval at 99%.

We then turned to the core of the analysis, by adding variables related to the pandemic phase into two stages. First, we added variables concerning the experience of the individual during the pandemic (see model 2 of Table 5). COVID-19 is a dummy variable which is equal to 1 when the individual or someone in her/his family tested positive to coronavirus, 0 otherwise (from our data, 7.6% of Italian population has experienced directly or indirectly negative health effects from coronavirus (see Table 3). This value seems to be coherent with official statistics. In fact, at June 2020, confirmed cases are nearly 0.5% of Italian population. This value must be times a factor of 7.3 if one considers only the close family network (see Istat, 2018). When one takes into account also the largest family network (second- and third-degree family members), one may reach percentages around 7–8% of Italian population). Moreover, we looked at the effect of lockdown introducing a dummy variable that is equal to 1 whenever the individual kept on going to the workplace during the lockdown period and is 0 in the opposite situation of people staying at home (of course, by comparing scores of individuals who experienced total lockdown with people that were forced to keep going to their workplace we greatly underestimate the effect of lockdown. Indeed, the second category of individuals suffered severe stress and anxiety from the particular situation and type of job they did (by definition public interest work in hospitals, supermarkets, pharmacies,…), so that are not able to compare lockdown with a “normal” situation). Results of model 2 show that COVID-19 is the variable that displays the greatest effect on the probability of falling into depression, anxiety and mental distress in general (0.733 and std. dev. 0.0562). To obtain a more analytical account of this effect, we calculated a probit model of scoring more than the cut-off (SMQF ≥ 12; see Table A.3) and then plot the probability of psychological distress as a function of age and COVID-19 (Fig. 3). The effect of this latter variable is huge and mostly doubles the probability of mental health distress at any age. Having experienced the virus within the family clearly makes the pandemic more vivid, it increases risk perception, generating worry for the loved one and for themselves which in turn affects mental health. Also, the fact of having experienced a complete lockdown shows a clear effect on depressive symptoms (the coefficient of “no lockdown” is equal to  − 0.123 with std. dev. 0.0551). However, the amplitude of this effect (and that of other variables) is of a different (much smaller) order, ranging between 1 and 3% (see Fig. 4) (however, as mentioned (see previous footnote), the coefficient of the variable “no lockdown” underestimates the effect of lockdown. Indeed, this result tells us that individuals who were forced to go to the workplace during this period and, presumably, suffered from stress and anxiety due to the situation and type of work they did, score significantly lower values of SMFQ than people under lockdown. Therefore, the effect of lockdown on depression may be significantly higher than that detected).

In the third step (models 3–6 of Table 5), we introduced variables related to the diffusion of the COVID-19 pandemic in the local area of residence of Italian citizens (either the county or the region) (at county level, the only available official statistics related to the number of COVID-19 confirmed cases. At regional level, there are other statistics such as the number of deaths and recovered individuals). In particular, we used the number of confirmed cases (at county level) and deaths (at a regional level) in June 2020, in terms of absolute and relative (as a % of local population) values. Indeed, these statistics were the most used by media outlets and public institutions to inform Italian citizens about the spread of the pandemic (in any case, the use of other local variables on the pandemic (recovered, intensive care, …) confirms results of Table 5). Results of models 3–6 of Table 5 confirm previous effects and show, interestingly, that the local diffusion of the pandemic does not affect the probability of scoring high values of SMFQ. This result is in line with previous results that investigated the dynamic evolution of TV viewership of Italian national and local news and showed that the attention of individuals to the pandemic is not related to the local spread but to the national diffusion^19^. In sum, results show that symptoms of mental distress like depression and anxiety are not connected with the local spread of the virus (but only with family cases), since they are widespread nationwide (and this may also be the effect of a national lockdown).

As a final step, we split the sample into two age groups (young adults and adults) and we ran count statistical models for the two groups, separately (see the Appendix for linear models, Table A.4). Results are reported in Table 6 (some variables (the professional ones—Unemployed, Lay-off and No lockdown—and Living alone) are not introduced in the model of younger individuals because they do not apply to them (or apply only to a very small part of them)) (in column 1 we report results for all individuals—as in model 2 of Table 5—, in column 2 those of young adults and in column 3 those of adults) and confirm previous outcomes for both age groups, in particular with regard to the significance and size of the effect of COVID-19 (whose coefficient is equal to 0.485—std. dev. 0.087—for young adults and to 0.77—std. dev. 0.0619). There are, however, three interesting and significative differences regarding younger individuals: (1) family wealth does not significantly affect negative mood of the youngest people, (2) the social context in terms of the size of the municipality where individuals live comes out to be less important for the youngest, and (3) age seems to display a U-inverted relation with score of negative mood. Indeed, this relation is positive for young people (0.101 and std. dev. 0.0143) and negative for older individuals (− 0.00954 and std. dev. 0.0013).

**Table 6:** Result of econometric models on the SMFQ score: young adults vs. adults.

Variables	(1)	(2)	(3)
	ALL	16–24	25+
	mfq	mfq	mfq
Gender (female)	0.238***	0.267***	0.241***
	(0.0302)	(0.0791)	(0.0328)
Age	− 0.00985***	0.101***	− 0.00954***
	(0.000838)	(0.0143)	(0.00113)
Unemployed	0.133***		0.125**
	(0.0503)		(0.0565)
Lay-off	0.316***		0.338***
	(0.0802)		(0.0933)
Poor	0.219***	0.143	0.227***
	(0.0425)	(0.187)	(0.0458)
Living alone	0.192***		0.192***
	(0.0409)		(0.0432)
Pop. municipality (log)	− 0.0203***	− 0.0191	− 0.0188**
	(0.00718)	(0.0193)	(0.00788)
COVID-19	0.733***	0.485***	0.770***
	(0.0562)	(0.0870)	(0.0619)
No lockdown	− 0.123**		− 0.113**
	(0.0551)		(0.0565)
Constant	2.054***	− 0.147	2.008***
	(0.0904)	(0.361)	(0.107)
Observations	6643	661	5982

^(^*^)^ Note: The estimates in the table refer to Poisson count models with double censoring (left at 0, right at 26), sample weights and robust standard errors. The dependent variable is the SMFQ score. For each variable, the coefficient, the standard error (in parentheses), and the level of significance are reported as follows: *** significant at 99%; ** significant at 95%.

## Discussion

To our knowledge, this is the first study exploring the effects of COVID-19 on mental health in a large, random and representative sample of the Italian population. Our results show higher scores of depressive symptoms indicative of depressive mood in females, younger adults, people reporting professional uncertainty and lower socio-economic status. A positive correlation was also found for individuals living alone, those who could not leave home for going to work, and people with a case of COVID-19 in the family. Interestingly, the region of residence was not a significant predictor of depressive symptoms.

Our findings highlight that the effect on mental health is related more broadly to the lockdown condition rather than the actual number of reported cases of deaths and infected (which were higher in the northern regions). The Short Mood and Feelings Questionnaire proved to be a reliable measure of depressive symptoms for both young and older adults, as shown by the separate analyses for age ranges 16–24 and 25+. The strength of the questionnaire lies in its easiness of administration and understanding, essential characteristics for detecting mental health’s effects during such an unprecedented situation.

Our research provides support to previous studies and extends the understanding of the psychological impact of the lockdown using a more reliable and scientifically sound method for sample’s selection. In line with our findings, previous studies on the psychological effects of COVID-19 also found that females are more affected by depression, anxiety, and distress^9,10,12^. Additionally, a recent review of the literature^20^ revealed that females are more likely to report depressive symptoms and to receive a diagnosis of depression.

The higher vulnerability of younger adults was observed in previous research as well in relation to anxiety and distress^11,12^. Our results complement this picture and reveal an association with negative symptoms indicative of depression. This result is of high importance when thinking about policy and health measures to be adopted to help younger generations to overcome the individual and social loss that they experienced during the pandemic.

Regarding work and financial conditions, our results confirm that professional uncertainty and low socio-economic status are related to mental health distress, as found in previous research^9^ for people with working discontinuity and financial struggles.

On the topic of household conditions, our findings highlight the role played by living alone in the experience of depressive symptoms. Despite new technologies aided in feeling closer to significant ones even from distance, the lack of social relations and daily gatherings significantly affected people’s mood. It is also worth noting that older individuals living by themselves could have been less familiar with the potential of new technologies, therefore limiting their chance to engage in virtual exchanges with loved ones.

Importantly, while previous studies revealed higher anxiety for individuals leaving their home to go to work^9,12^, our research found lower depression in those people who kept going physically to the workplace. This finding shows that, even if going out triggered anxiety and fear of getting infected, keeping a working routine helped to feel less depressed and lonely.

Lastly, as previous research has shown^9,11,12^, having a case of COVID-19 in the family had an impact on reported depressive symptoms. In particular, our study shows that the effect of this factor is huge and mostly doubles the probability of psychological distress at any age. Also, the fact of having experienced a complete lockdown also shows a clear and significant effect, ranging at least between 1 and 3%, on depressive symptoms. Overall, this negative experience could amplify the feelings of fear, anxiety, stress and enhance the perceived risk on the probability of infection and the potentially fatal consequences derived from catching the virus.

It is crucial to remark that, other things being equal (in particular, the eventual presence of a COVID-19 case in the family), no differences were found in relation to the region of residence, while previous studies^10,11^ found more sleep disturbances and state of anxiety in Northern Italy. Our study, which is statistically grounded, reveals that feelings of anxiety and depression were spread in the whole country (this effect is clearly linked to the very nature of the phenomenon under investigation (i.e. a pandemic) that can potentially spread everywhere and is therefore likely to generate anxiety and depression even in places where it has not yet arrived (or will never arrive)).

Despite the strengths of the present research, some limitations are worth mentioning. First, the cross-sectional design does not allow to draw conclusions on causal inferences between the lockdown and the depressive symptoms. Second, the use of self-report measures can lead to overestimation of the degree of psychological distress. Third, other mental health conditions such as anxiety, stress and quality of life were not assessed, being depression the focus of the study.

As highlighted by some researchers^21^, it is likely that the prevalence of clinically relevant numbers of people with anxiety, depression, and engaging in harmful behaviours (such as suicide and self-harm) will increase. The potential consequences of an economic recession on mental health may be acute on people directly affected by COVID-19 and their caregivers. Data on the severe acute respiratory syndrome epidemic in 2003 reported a 30% increase in suicide in those aged 65 years and older; around 50% of recovered patients remained anxious; and 29% of health-care workers experienced probable emotional distress. Post-traumatic stress disorder and depression were also reported among patients who survived severe and life-threatening illness^21^.

## Conclusions and future work

The present research stresses the need to take into account the psychological consequences of the COVID-19 pandemic and lockdown, aiming at the implementation of a holistic approach that considers both physical and mental health and well-being. The society as a whole, and in particular vulnerable groups such as children, older adults, people with existing mental health disorders, and front-line health-care workers, call for support to overcome this difficult time. Next months will be characterised by uncertainty, financial insecurity and worry, therefore it is pivotal to provide help through mental health care which could also make use of the Telemedicine, e.g., telehealth and app tools^6^. Indeed, online interactions can promote a sense of connection and improve psychological well-being^22^.

Future studies should consider the long-term effects of the pandemic on mental health, adopting a longitudinal design to measure change over time. Additional work should aim at comparing the experiences of the different countries affected by the pandemic in order to understand the size of the psychological impact and the potential risk and protective factors. Importantly, the data on people who seek for mental health assistance should closely monitored to prevent a second pandemic of psychological distress.

## Supplementary Information

Supplementary Information.
