# Supplementary material for: Depressive symptoms in response to COVID-19 and lockdown: a cross-sectional study on the Italian population
Source: Sci Rep. 2020 Dec 31;10:22457. doi: 10.1038/s41598-020-79850-6 (PMC7775443; doi:10.1038/s41598-020-79850-6)
Supplement: Supplementary file 1 — Supplementary Information. [file 41598_2020_79850_MOESM1_ESM.docx]

**Depressive Symptoms in Response to COVID-19 and Lockdown: A Cross-sectional Study on the Italian Population**

Marco Delmastro ^a^ and Giorgia Zamariola ^b^

^a^ Autorità per le garanzie nelle comunicazioni, Via Isonzo 21/b Roma – Centro Direzionale/Isola B5 Napoli

^b^ Alma Mater Studiorum – Università di Bologna, Via Zamboni, 33, 40126 Bologna

**Corresponding Author:**

Marco Delmastro

m.delmastro@agcom.it

**Appendix**

**Table A.1: Summary statistics of independent variables: young adults (16-24) and adults (25+)**

| **Variable** | **Description** | **Young adults (16-24)** | | **Adults (25+)** | |
| --- | --- | --- | --- | --- | --- |
|  |  | **N.** | **Avg.** | **N.** | **Avg.** |
| Gender (female) | 1 if female; 0 otherwise | 666 | .478 | 6,026 | .522 |
| Age | Age (number of years) | 666 | 20.299 | 6,026 | 53.697 |
| Unemployed | 1 if unemployed; 0 oth. | 666 | .093 | 6,026 | .074 |
| Lay-off | 1 if a lay-off; 0 otherwise | 666 | .017 | 6,026 | .031 |
| Poor | 1 if poor^(*)^; 0 otherwise | 666 | .061 | 6,026 | .131 |
| Living alone | 1 if she/he lives alone; 0 otherwise | 666 | .058 | 6,026 | .159 |
| COVID-19 | 1 if a COVID-19 case in her/his family; 0 otherwise | 661 | .088 | 5,982 | .075 |
| No lockdown | 1 if he/she has kept working from workplace (during lockdown); 0 if she/he experienced home lockdown | 666 | .025 | 6,026 | .118 |

^(*)^ “Poor” is defined from a 5-point Likert scale as the worst economic condition, in which the individual experiences severe economic difficulties, lacking sufficient money to live at a normal standard.

**Table A.2: Result of linear models (OLS) on the SMFQ score**

|  | (1) | (2) | (3) | (4) | (5) | (6) |
| --- | --- | --- | --- | --- | --- | --- |
|  | regr_1 | regr_2 | regr_3 | regr_4 | regr_5 | regr_6 |
| VARIABLES | Mfq | mfq | mfq | mfq | mfq | Mfq |
|  |  |  |  |  |  |  |
| Gender (female) | 0.960*** | 1.011*** | 1.011*** | 1.012*** | 1.013*** | 1.012*** |
|  | (0.147) | (0.144) | (0.144) | (0.144) | (0.144) | (0.144) |
| Age | -0.055*** | -0.049*** | -0.049*** | -0.049*** | -0.049*** | -0.049*** |
|  | (0.004) | (0.004) | (0.004) | (0.004) | (0.004) | (0.004) |
| Unemployed | 1.004*** | 0.758** | 0.757** | 0.737** | 0.733** | 0.738** |
|  | (0.326) | (0.318) | (0.319) | (0.319) | (0.319) | (0.319) |
| Lay-off | 2.690*** | 2.170*** | 2.171*** | 2.173*** | 2.162*** | 2.170*** |
|  | (0.502) | (0.475) | (0.475) | (0.476) | (0.476) | (0.476) |
| Poor | 1.101*** | 1.174*** | 1.173*** | 1.157*** | 1.165*** | 1.163*** |
|  | (0.243) | (0.240) | (0.240) | (0.240) | (0.240) | (0.240) |
| Living alone | 0.949*** | 1.056*** | 1.057*** | 1.065*** | 1.067*** | 1.066*** |
|  | (0.220) | (0.219) | (0.219) | (0.220) | (0.219) | (0.219) |
| Pop. municipality (log) | -0.128*** | -0.108*** | -0.107*** | -0.113*** | -0.110*** | -0.111*** |
|  | (0.0377) | (0.0368) | (0.0379) | (0.0372) | (0.0369) | (0.0370) |
| COVID-19 |  | 4.885*** | 4.886*** | 4.909*** | 4.906*** | 4.903*** |
|  |  | (0.364) | (0.365) | (0.365) | (0.364) | (0.364) |
| No lockdown |  | -0.694*** | -0.695*** | -0.697*** | -0.703*** | -0.698*** |
|  |  | (0.234) | (0.234) | (0.233) | (0.234) | (0.234) |
| # COVID-19 cases (county) |  |  | -1.68e-06 |  |  |  |
|  |  |  | (1.20e-05) |  |  |  |
| % COVID-19 cases pop. county |  |  |  | -25.36 |  |  |
|  |  |  |  | (20.63) |  |  |
| # COVID-19 deaths (region) |  |  |  |  | -1.85e-05 |  |
|  |  |  |  |  | (1.25e-05) |  |
| % COVID-19 deaths pop region |  |  |  |  |  | -145.5 |
|  |  |  |  |  |  | (126.3) |
| Constant | 8.427*** | 7.568*** | 7.563*** | 7.712*** | 7.658*** | 7.673*** |
|  | (0.472) | (0.476) | (0.478) | (0.492) | (0.481) | (0.486) |
|  |  |  |  |  |  |  |
| Observations | 6,692 | 6,643 | 6,643 | 6,643 | 6,643 | 6,643 |
| R-squared | 0.061 | 0.117 | 0.117 | 0.117 | 0.117 | 0.117 |

^(*)^ Note: The estimates in the table refer to linear (OLS) models with sample weights and robust standard errors. The dependent variable is the SMFQ score. The models are estimated using the constant. For each variable, the coefficient, the standard error (in parentheses), and the level of significance are reported as follows: *** significant at 99%; ** significant at 95%.

**Table A.3: Result of a Probit model on the probability of depression (SMFQ score ≥ 12)**

|  | (1) |
| --- | --- |
| VARIABLES | mfq_d |
|  |  |
| Gender (female) | 0.232*** |
|  | (0.0450) |
| Age | -0.0123*** |
|  | (0.00132) |
| Unemployed | 0.0614 |
|  | (0.0855) |
| Lay-off | 0.514*** |
|  | (0.119) |
| Poor | 0.241*** |
|  | (0.0665) |
| Living alone | 0.321*** |
|  | (0.0598) |
| Pop. municipality (log) | -0.0317*** |
|  | (0.0110) |
| COVID-19 | 0.927*** |
|  | (0.0704) |
| No lockdown | -0.178** |
|  | (0.0779) |
| Constant | -0.438*** |
|  | (0.139) |
|  |  |
| Observations | 6,643 |

^(*)^ Note: The estimates in the table refer to a Probit model with sample weights and robust standard errors. The dependent variable is 1 if the SMFQ score is ≥1; and is 0 is <12, where scoring a 12 or higher may indicate the presence of depression in the respondent (see Thabrew et al., 2018). For each variable, the coefficient, the standard error (in parentheses), and the level of significance are reported as follows: *** significant at 99%; ** significant at 95%.

**Table A.4: Result of linear models (OLS) on the SMFQ score: young adults vs. adults**

|  | (1) | (2) | (3) |
| --- | --- | --- | --- |
|  | ALL | 16-24 | 25+ |
| VARIABLES | mfq | Mfq | mfq |
|  |  |  |  |
| Gender (female) | 1.011*** | 1.475*** | 0.949*** |
|  | (0.144) | (0.498) | (0.149) |
| Age | -0.0489*** | 0.584*** | -0.0428*** |
|  | (0.00431) | (0.0779) | (0.00529) |
| Unemployed | 0.758** |  | 0.643 |
|  | (0.318) |  | (0.336) |
| Lay-off | 2.170*** |  | 2.290*** |
|  | (0.475) |  | (0.499) |
| Poor | 1.174*** | 0.288 | 1.247*** |
|  | (0.240) | (1.096) | (0.245) |
| Living alone | 1.056*** |  | 0.993*** |
|  | (0.219) |  | (0.219) |
| Pop. municipality (log) | -0.108*** | -0.165 | -0.0960** |
|  | (0.0368) | (0.135) | (0.0382) |
| COVID-19 | 4.885*** | 4.553*** | 4.942*** |
|  | (0.364) | (0.879) | (0.394) |
| No lockdown | -0.694*** |  | -0.601** |
|  | (0.234) |  | (0.240) |
| Constant | 7.568*** | -4.162 | 7.080*** |
|  | (0.476) | (2.292) | (0.526) |
|  |  |  |  |
| Observations | 6,643 | 661 | 5,982 |
| R-squared | 0.117 | 0.142 | 0.111 |

^(*)^ Note: The estimates in the table refer to linear (OLS) models with sample weights and robust standard errors. The dependent variable is the SMFQ score. The models are estimated using the constant. For each variable, the coefficient, the standard error (in parentheses), and the level of significance are reported as follows: *** significant at 99%; ** significant at 95%.
